# Knowledge, attitudes, and self-reported practices regarding cholera among six MENA countries following cholera outbreaks in the region

**DOI:** 10.1186/s12889-025-21731-6

**Published:** 2025-02-18

**Authors:** Salma A. Bekhit, Rayane Rafei, Fatma Elnaggar, Omar Zain AL-Sakkaf, Hussein Kamal Seif, Dana Samardali, Yara Turkmani Alabead, Mohammed Osman Omer Sanosi, Marwa Shawky Abdou, Eman H. Elbanna, Doaa Mahmoud Khalil

**Affiliations:** 1 Department of Environmental Health, High Institute of Public Health, Alexandria University, Alexandria, 21561 Egypt; 2 Laboratoire Microbiologie Santé et Environnement (LMSE), Doctoral School of Science & Technology, Faculty of Public Health, Lebanese University, Tripoli, 1300 Lebanon; 3 Health Administration and Behavioral Sciences Department, High Institute of Public Health, Alexandria University, Alexandria, 21561 Egypt; 4 Public Health and Community Medicine Department, Faculty of Medicine and Health Sciences, Aden University, Aden, Yemen; 5 Zagazig University Hospitals, Zagazig University, Al Sharqia, Egyp Egypt; 6 Faculty of Medicine, Yarmouk University, Irbid, Jordan; 7 Damascus Hospital, Damascus, Syria; 8 Faculty of Public Health, University of Shendi, Shendi, Sudan; 9 Department of Epidemiology, High Institute of Public Health, Alexandria University, Alexandria, 21561 Egypt; 10 Health Management, Planning and Policy Department, High Institute of Public Health, Alexandria University, Alexandria, 21561 Egypt; 11 Public Health and Community Medicine Department, Faculty of Medicine, Beni-Suef University, Beni-Suef, Egypt

**Keywords:** Cholera outbreak, MENA countries, Knowledge, Attitude, Practices

## Abstract

**Background:**

Cholera persists as a global public health threat, endangering the lives of vulnerable societies including the MENA region where many countries are facing recent cholera outbreaks. The present study aimed to characterize the knowledge, attitude, and practices status related to cholera in six MENA countries in the MENA region.

**Methods:**

A cross-sectional study was conducted using a structured, validated questionnaire and distributed across different social media platforms in Egypt, Sudan, Jordan, Syria, Lebanon, and Yemen between December 2022 and January 2023. Univariate and multivariate analyses were used to determine factors associated with knowledge, attitudes, and practices related to cholera.

**Results:**

A total of 2971 participants were included in the study, of which 62.5% were females; with a mean age of 34.8 ± 12.3 years; 85.4% heard about cholera; and 1.9% experienced cholera infection during cholera outbreaks in their countries. Among those who heard about cholera, 50.7% had adequate knowledge, 67.3% had desirable attitudes, and 50.3% reported good practices. Multivariate analysis revealed that being older, highly educated, employed, working in the medical field, and living in an outbreak country were the significant predictors affecting good knowledge. Additionally, good attitudes were significantly increased by older ages, females, those working in the medical sector, and those living in an outbreak country. Whereas working in the medical sector and having a larger number of people living in the same house significantly decreased the practice scores.

**Conclusions:**

Raising community awareness about fecal-oral diseases transmitted via contaminated food or water, such as cholera, is crucial. This can be achieved by organizing targeted awareness campaigns for the whole community. Furthermore, mandatory educational workshops and programs for medical professionals are essential, as they serve as role models for the community.

**Supplementary Information:**

The online version contains supplementary material available at 10.1186/s12889-025-21731-6.

## Background

Cholera, caused by the Gram-negative bacterium *Vibrio cholerae* and transmitted via the fecal-oral route through contaminated water or food, is a highly virulent diarrheal disease primarily linked to serogroups O1 and O139 [1, 2]. While most infections are asymptomatic or mild, symptomatic cases can result in severe dehydration due to profuse watery diarrhea and vomiting, potentially fatal if not appropriately treated [2]. Annually, cholera affects an estimated 1.3 to 4 million people and causes 21,000 to 143,000 deaths [3]. Short-term management of cholera involves rehydration salts and antibiotics, with the World Health Organization (WHO) recommending oral cholera vaccines (OCVs) in hotspot areas [4, 5]. Long-term prevention necessitates interdisciplinary collaboration in education, sanitation, diagnostics, surveillance, and outbreak preparedness [6–8].

Amidst the waves of the COVID-19 pandemic, healthcare systems were overwhelmed, diverting resources and attention away from other important endemic diseases like cholera [9, 10]. The Middle East and North Africa (MENA) region has not been spared, with many countries like Yemen, Syria, and Lebanon reporting cholera cases in the last decade [11], including, recording suspected/confirmed cases in 2023 [12]. In 2017, WHO recorded 1.2 million cholera cases and 5654 mortalities worldwide, with 84% and 41% of cholera-attributed cases and deaths reported only from Yemen, marking it as the largest cholera epidemic in history [13, 14].

Yemen’s ongoing civil war since 2014 has devastated sanitation facilities and healthcare systems, displaced millions, along with environmental changes, have greatly contributed to the spread of cholera [13, 15]. Similarly, Syria’s healthcare infrastructure suffered after the 2011-armed conflict, which led to the mass exodus of millions including healthcare workers, and the lack of clean water, sanitation, and hygiene. On September 10, 2022, the Syrian Health Ministry declared a cholera outbreak, further worsened by the February 6, 2023, earthquake in Northwest Syria [16, 17]. Meanwhile, Lebanon has welcomed many Syrian refugees, burdening the already economically suffering country [18]. On 6 October 2022, Lebanon reported its first cholera cases in nearly three decades, amounting to 671 confirmed cases by January 5, 2023 [19]. The outbreak began with a Syrian index case in North Lebanon and was likely driven by the influx of Syrian refugees and the shared Orontes River [20].

Egypt was selected for this study due to its large refugee population and recently deteriorated economic conditions, which have further limited access to education, hygiene, water, and healthcare for both refugees and local residents [21]. Likewise, Jordan is drastically affected by the Syrian war and is hosting the second-largest number of Syrian refugees per capita globally, alongside others from the MENA region [22]. Meanwhile, Sudan’s ongoing armed conflict has displaced over 2.8 million people and led to a breakup in the health system and a lack of essential services [23, 24]. These factors place Egypt, Jordan, and Sudan at high risk for cholera outbreaks.

Several studies have assessed knowledge, attitudes, and practices (KAP) related to cholera conducted in outbreak countries, neglecting cholera-free countries in hotspot regions [25–28]. Identifying gaps in cholera-related knowledge, attitudes, and practices in these cholera-free areas is vital for developing prevention and control strategies and tailoring awareness and education programs to avoid severe cholera consequences. Therefore, the current study aimed to evaluate cholera-related KAP among populations in six MENA countries, of which three countries are cholera-free and three are experiencing cholera outbreaks, using a developed and validated questionnaire.

## Methods

### Study design and study setting

Using the snowball sampling technique, a cross-sectional online survey was conducted using Google Forms in six MENA countries (Egypt, Sudan, Jordan, Syria, Lebanon, and Yemen) from 15 December 2022 to 12 January 2023. After explaining the research objectives to the participants, they were asked to participate in the study via various social media platforms such as WhatsApp, Facebook, Telegram, and LinkedIn. Those interested in participating in the study were asked to fill out the online form of a self-administrated questionnaire and to share it with their social contacts.

### Study participants and sample size

We recruited participants from Egyptian, Sudanese, Jordanian, Syrian, Lebanese, and Yemeni populations who were 18 years or above and had computer devices or smartphones with internet access. Since there were no published studies in most of the studied countries to assess the knowledge of the studied populations about cholera, we supposed that 50% of the participants in each country had good knowledge about cholera. The sample size was calculated using Epi Info stat-calc for the population survey at a 99.99% confidence level, 5% acceptable margin of error, 1 design effect, and 50% expected frequency (of good knowledge) in 6 clusters. The minimum sample size for this study was estimated to be 253 participants in each cluster with a total sample of 1512. The sample size was almost doubled to overcome the selection bias.

### Data collection tools and scoring system

The language used in this study was Standard Arabic since it is the official language in all the chosen six MENA countries. A questionnaire of four self-reported domains was developed to collect data from participants in the six MENA countries [Yemen, Syria, and Lebanon (cholera-outbreak countries), and Egypt, Sudan, and Jordan (cholera-free countries)]. The first domain collected data about (i) sociodemographic characteristics, baseline characteristics, and health conditions (i.e., country of residence, nationality, sex, age, marital status, education, employment status, profession, monthly income, having children, living with an elderly person, number of people living within the same household, history of chronic diseases, participation in food handling/ preparation, self-assessment of food safety knowledge, source of knowledge related to food safety) and (ii) general knowledge about cholera (heard about cholera before, heard about the recent cholera outbreaks in some Arabic countries and the source of this information, previous cholera infection during the outbreak, need for hospitalization, cholera infection among relatives, mortalities among family members due to cholera). The second domain included 21 items that assessed the knowledge of the cause, symptoms, treatment, vaccination, and risk factors of cholera. Responses to the knowledge domain were categorized into (yes, no, do not know). Regarding the scoring of the knowledge domain, each correct answer was given 1 mark, while the wrong answer and do not know were considered zero; thus, the total score ranged from zero to 21 marks. We categorized the knowledge score by median (15) into optimal knowledge (> 15) and sub-optimal knowledge (≤ 15). In the third domain, questions asked about attitudes toward susceptibility to getting infected with cholera, cholera severity, getting a vaccination, side effects of cholera vaccine, having a role in cholera prevention, and traveling to epidemic areas; this domain consisted of 6 items. The responses to the questions of the attitude domain were recorded in the form of a 3-point Likert scale ranging from “disagree” to “agree”. For positive statements, “agree” was given (3) points, while “disagree” was given (1) point. Reverse coding was used for negative statements. We categorized it into 2 categories by its median (2.3); respondents who scored above the median were considered as having a good attitude (toward agreement) and less than or equal to the median score as a poor attitude (toward disagreement). Finally, the fourth domain evaluated the participants’ hand hygiene, sanitation, food, and water safety practices, containing 18 items. The responses to the practice domain were organized into a 5-point Likert scale as follows: never (1), rarely (2), sometimes (3), often (4), and always (5). The practice score ranged from 1 to 5, and we categorized it by its median (4.2) into suboptimal practice (≤ 4.2) and optimal practice (> 4.2).

The questionnaire was revised and evaluated by a panel of experts with experience in the field of infectious diseases, food safety, public health, and questionnaire design. The panel consisted of six experts (S.A.B, R.R, D.M.K, F.E, M.S.A, E.H.E). Before administrating the questionnaire, trained research team members conducted cognitive interviews among 40 participants from the six studied countries, who were then excluded from the main study and the subsequent data analysis. These interviews were conducted to ensure language clarity, ease of comprehension, good readability, and cultural appropriateness of the questions. The questionnaire took 10–15 min to complete. The piloted participants reported no difficulties; thus, no further changes were made. Cronbach’s alphas were calculated for the questionnaire’s second, third, and fourth domains to assess their internal consistency. No interclass correlation was detected in the initial pilot study; therefore, no components were deleted from the original version. Cronbach’s alpha value of the second domain (knowledge) was 0.727, that of the third domain (attitude) was 0.523, and that of the fourth domain (practices) was 0.924. This means that the knowledge and practices domains had an acceptable validity as Cronbach’s Alpha value was > 0.6, while the attitudes domain had a value of < 0.6.

### Statistical analysis

The data were managed and analyzed using SPSS software version 26.0 (Chicago, USA). Scale variables were presented using mean ± standard deviation (SD) for the normally distributed variables. Categorical variables were presented as frequencies and percentages. The association between categorical variables was assessed using the Chi-Square test. To compare the relationship between categorical and scale, an independent t-test was done. Multivariate binary logistic regression analysis was conducted on the factors that showed statistical significance in the earlier analysis to illustrate those associated with the probability of optimal knowledge, attitudes, and practice. Statistical significance was defined as a *p*-value of < 0.05 and a proportion odds ratio (POR) bigger than one with 95% CI that didn’t cross the null was considered a significant factor associated with good KAP.

## Results

### Study sample characteristics

**Fig. 1:**
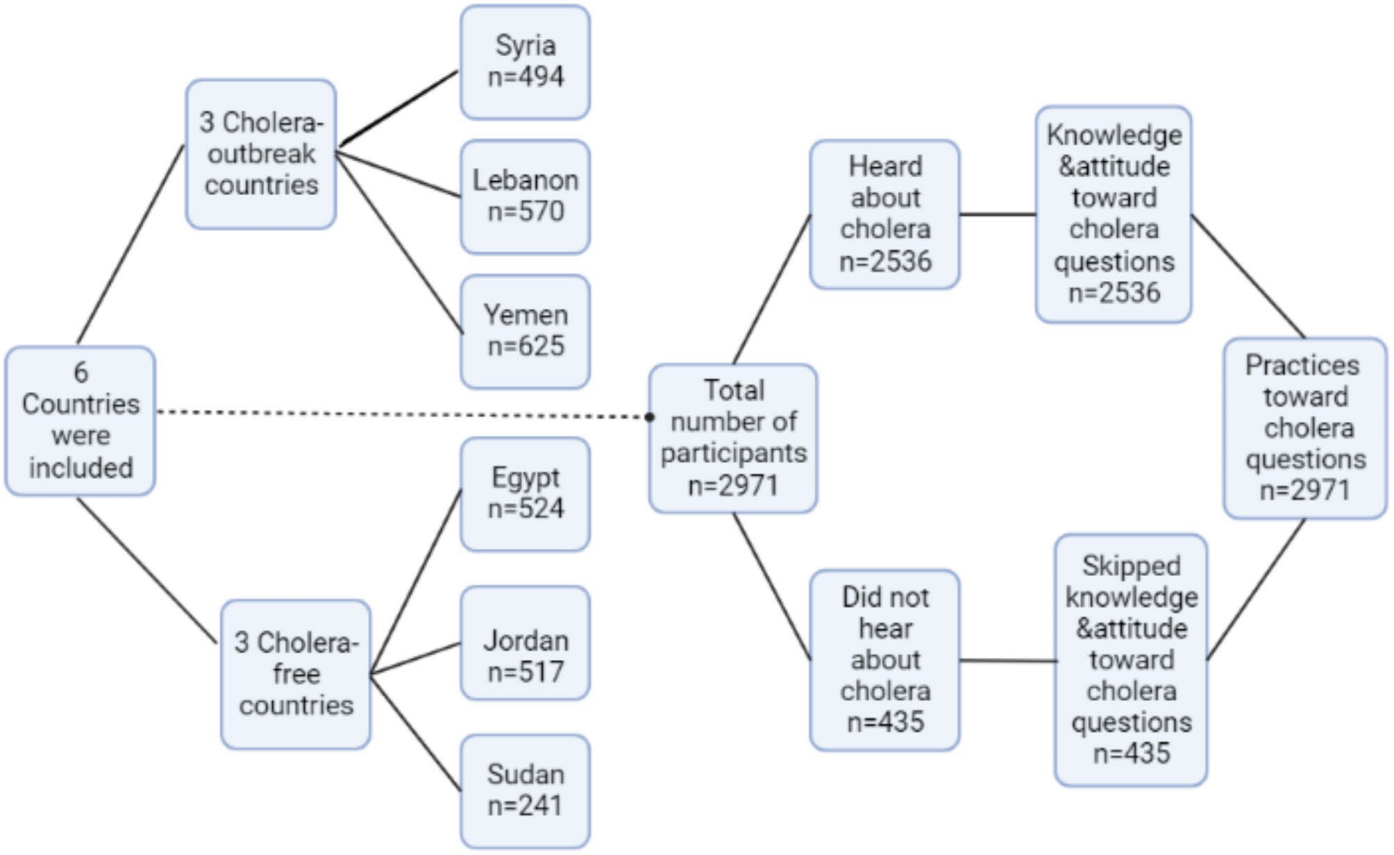
Flowchart of the data collection procedure

A total of 2971 participants from six Arabic countries were included in this study, with 21% of participants being from Yemen, 19.2% from Lebanon, 17.6% from Egypt, 17.4% from Jordan, 16.6% from Syria, 8.1% from Sudan (Fig. 1). Collecting data from Sudan was very difficult because of the war situation that led to internet and communications outages, leading to the drop of 12 persons from the required sample size of Sudan. The mean age of respondents was 34.8 ± 12.3 years with 62.5% (*n* = 1856) being females and 55.9% (*n* = 1661) being married. More than half of the participants (55.8%, *n* = 1657) had a university degree and 63.2% (*n* = 1878) respondents were employed with 41.3% (*n* = 776) of them working inside the medical field. The income for 44.4% (*n* = 1319) of participants was enough for them, 49.5% (*n* = 1470) of respondents had children, 56.6% (*n* = 1682) lived with an elderly relative, and 36.7% (*n* = 1151) had chronic diseases. Most of the participants (76.8%, *n* = 2281) had either very good or good self-assessment of food safety knowledge, with 43.1% (*n* = 1280/2971) having multiple sources of information regarding food safety (including internet, social media platforms, and family/friends) (Additional file 1: Table S1).

The information source of participants and their previous experience with cholera were also assessed (Additional file 1: Table S2). Most participants (85.4%, *n* = 2536) heard about cholera disease, of which 85.9% (*n* = 2179) heard about the recent cholera outbreaks in Arab countries with their main sources of information being social media (24.6%, *n* = 535), healthcare providers (10.8%, *n* = 235) and TV or radio (10.1%, *n* = 220), respectively. Nearly 42 out of 2179 (1.9%) participants got infected during the cholera outbreaks in the affected countries, with only 5 of them (11.9%) required to be hospitalized. Out of 2179 participants who heard about the recent Arabian cholera outbreak, 7.3% (*n* = 160) of their relatives got infected with cholera, of which 38.1% (*n* = 61/160) needed hospitalization, and 18.1% (*n* = 29/160) died due to cholera. When participants were asked what they would do if they got infected with cholera, 52.8% of them (*n* = 1570) answered that they would go to a health facility and take the prescribed medication, and 7.9% of them (*n* = 235) would prepare oral rehydration solutions (ORS) at home.

### Knowledge, attitudes, and practices of the participants toward cholera disease and preventive measures

The participants’ answers regarding their knowledge and attitudes toward cholera are summarized in Additional file 1: Table S3. Only 2536 participants who answered that they heard about cholera were allowed to answer the knowledge and attitudes questions, while the others skipped these parts and answered the part related to the practices (Fig. 1). The majority of participants answered the questions of general knowledge correctly, except for those regarding the non-symptomatology of most cholera cases and the route of cholera vaccine administration, where only 5.1% (*n* = 130) and 38.9% (*n* = 986) answered these questions correctly, respectively. Questions regarding the modes of transmission and symptoms were correctly answered by most of the participants. The mean knowledge score was 13.7 ± 4.1, with 50.7% (*n* = 1287/2536) having a good level of knowledge (scored more than 15.0). Regarding attitudes, the participants scored an average of 2.4 ± 0.3 with 67.3% having desirable attitudes. Out of 2536 participants, 89.2% believed that cholera is a severe health problem, 70.8% that the cholera vaccine could protect them, 17.0% that vaccination had no serious side effects, 47.3% that they were highly susceptible to cholera, 39.7% that they had no role in preventing its spread in the community, and 55.9% that traveling to an area having cholera outbreak will increase risk of acquiring the disease.

Participants were asked about hand hygiene practices (Additional file 1: Table S4). Most of the study participants (*n* = 2971) wash their hands regularly, and in different circumstances, thus leading to a total hand hygiene practices score of 4.1 ± 1.0. Food and water safety practices were also retrieved to be practiced by most of the participants giving a total food and water safety practices score of 3.9 ± 1.0. and a percentage of 50.3% of participants with good practices (Additional file 1: Table S4). Besides, the sources, methods of treatment, and quality testing of the drinking water were also surveyed among the participants (Additional file 1: Table S5-S9). Only about 37.1% (*n* = 1102) of participants use public water networks as the main water source, while the others rely on other water sources. In addition, 27.9% (*n* = 826) do not treat water before usage, and 19.6% (*n* = 583) test the water quality before using it.

### Comparison of knowledge between different variables

Respondents with good knowledge relative to those with poor knowledge had significantly higher mean age, earned a university and above degree, were employed, worked in the medical sector, lived in cholera-outbreak countries (Yemen, Syria, and Lebanon), had children, did not live with elderly relatives (Table 1). The mean total attitudes score was significantly higher among respondents with good knowledge than those with poor knowledge (2.4 ± 0.3 vs. 2.3 ± 0.3, respectively, *p* < 0.001). Similarly, the mean total practices score was significantly related to good knowledge compared to those with poor knowledge (4.1 ± 1.0 vs. 3.8 ± 1.04, respectively, *p* < 0.001) (Table 1).

**Table 1:** Baseline characteristics of the studied participants associated with poor and good cholera knowledge

Characteristics	Total	Poor Knowledge (≤ 15) (*N* = 1249)	Good Knowledge (> 15) (*N* = 1287)	*p*
**Age**		32.9 ± 11.6	36.6 ± 12.9	< 0.001*
**Sex**				0.442
Male	951	459 (48.3)	492 (51.7)	
Female	1585	790 (49.8)	795 (50.2)	
**Marital status**				0.117
Un-married	1155	588 (50.9)	567 (49.1)	
Married	1381	661 (47.9)	720 (52.1)	
**Educational level**				0.024*
Secondary or less	471	254 (53.9)	217 (46.1)	
University and above	2065	995 (48.2)	1070 (51.8)	
**Employment status**				< 0.001*
Unemployed/Retired	899	492 (54.7)	407 (45.3)	
Employed	1637	757 (46.2)	880 (53.8)	
**Working field (***n* = **1637)**				< 0.001*
Non-Medical sectors	892	490 (54.9)	402 (45.1)	
Medical sector	745	267 (35.8)	478 (64.2)	
**Residence**				< 0.001*
Cholera-free countries	889	620 (69.7)	269 (30.3)	
Outbreak countries	1647	629 (38.2)	1018 (61.8)	
**Income**				0.085
Not enough	1922	928 (48.3)	994 (51.7)	
Enough	614	321 (52.3)	293 (47.7)	
**Having children**				0.002*
Yes	1308	606 (46.3)	702 (53.7)	
No	1228	643 (52.4)	585 (47.6)	
**Living with an elderly relative**				< 0.001*
Yes	1412	744 (52.7)	668 (47.3)	
No	1124	505 (44.9)	619 (55.1)	
**Number of family members (including the participant)**		4.8 ± 2.1	4.9 ± 2.2	0.082
**Prepare/help in preparing food**				0.561
Yes	1881	920 (48.9)	961 (51.1)	
No	655	329 (50.2)	326 (49.8)	
**Chronic diseases**				0.096
Yes	2399	1191 (49.6)	1208 (50.4)	
No	137	58 (42.3)	79 (57.7)	
**Total attitudes score**		2.3 ± 0.31	2.4 ± 0.3	< 0.001*
**Total practices score**		3.8 ± 1.04	4.1 ± 1.0	< 0.001*

### Comparison of attitudes between different variables

Good cholera attitudes among respondents were significantly associated with a higher mean age; being male, married, employed, and without chronic disease; working inside the medical sector; having children; and not living with elderly relatives. Living in outbreak countries appeared to be linked with good attitudes, but this association was not statistically significant (68.5% vs. 64.9%, *p* = 0.062). A lower number of family members was associated with good attitudes compared to a higher family member number, *p* = 0.02 (Table 2).

**Table 2:** Baseline characteristics of the studied participants associated with poor and good cholera attitudes (*N* = 2536)

Characteristics	Total	Poor attitudes (≤ 2.3) (*N* = 830)	Good attitudes (> 2.3) (*N* = 1706)	*p*
**Age**		33.0 ± 12.0	35.7 ± 12.5	< 0.001*
**Sex**				0.006*
Male	951	280 (29.4)	671 (70.6)	
Female	1585	550 (34.7)	1035 (65.3)	
**Marital status**				0.007*
Un-married	1155	410 (35.5)	745 (64.5)	
Married	1381	420 (30.4)	961 (69.6)	
**Educational level**				0.436
Secondary or less	471	147 (31.2)	324 (68.8)	
University and above	2065	683 (33.1)	1382 (66.9)	
**Employment status**				< 0.001*
Unemployed/Retired	899	337 (37.5)	562 (62.5)	
Employed	1637	493 (30.1)	1144 (69.9)	
**Working field (***n* = **1637)**				0.008*
Non-Medical sectors	892	293 (32.8)	599 (67.2)	
Medical sector	745	200 (26.8)	545 (73.2)	
**Residence**				0.062
Cholera-free countries	889	312 (35.1)	577 (64.9)	
Outbreak countries	1647	518 (31.5)	1129 (68.5)	
**Income**				0.894
Not enough	1922	613 (31.9)	1309 (68.1)	
Enough	614	217 (35.3)	397 (64.7)	
**Having children**				0.002*
Yes	1308	391 (29.9)	917 (70.1)	
No	1228	439 (35.7)	789 (64.3)	
**Living with an elderly relative**				0.014*
Yes	1412	491 (34.8)	921 (65.2)	
No	1124	339 (30.2)	785 (69.8)	
**Number of family members (including the participant)**		5.0 ± 2.2	4.8 ± 2.1	0.020*
**Prepare/help in preparing food**				0.672
Yes	1881	620 (33.0)	1261 (67.0)	
No	655	210 (32.1)	445 (67.9)	
**Chronic diseases**				0.003*
Yes	2399	801 (33.4)	1598 (66.6)	
No	137	29 (21.2)	108 (78.8)	

### Comparison of practices between different variables

Good practices were significant among females, unmarried, unemployed/retired, working outside medical sectors, without enough income, not having children, not living with an elderly person, having a low family member number, and respondents with multiple information sources (Table 3). Respondents with excellent, very good, and good self-assessment of food safety knowledge were significantly associated with good practices (Table 3). Practices score showed a significant positive weak correlation with knowledge score (*r* = 0.1, *p* < 0.001) and attitudes score (*r* = 0.075, *p* < 0.001). Also, the attitudes score had a significant positive weak correlation with the knowledge score (*r* = 0.251, *p* < 0.001) (Additional file 1).

**Table 3:** Baseline characteristics of the studied participants associated with poor and good cholera practices (*N* = 2971)

Characteristics	Total	Poor practices (≤ 4.2) (*N* = 1476)	Good practices (> 4.2) (*N* = 1495)	*p*-value
**Age** (mean ± SD)		34.89 ± 12.0	34.6 ± 12.7	0.605
**Sex**				< 0.001*
Male	1115	631 (56.6)	484 (43.6)	
Female	1856	845 (45.5)	1011 (54.5)	
**Marital status**				0.001*
Un-married	1310	606 (46.3)	704 (53.7)	
Married	1661	870 (52.4)	791 (47.6)	
**Educational level**				0.5
Secondary or less	629	320 (50.9)	309 (49.1)	
University and above	2342	1156 (49.4)	1186 (50.6)	
**Employment status**				0.005*
Unemployed/Retired	1093	506 (46.3)	587 (53.7)	
Employed	1878	970 (51.7)	908 (48.3)	
**Working field (***n* = **1878)**				0.018*
Non-medical sector	1102	544 (49.4)	558 (50.6)	
Medical sector	776	426 (54.9)	350 (45.1)	
**Residence**				0.087
Cholera-free countries	1282	660 (51.5)	622 (48.5)	
Outbreak countries	1689	816 (48.3)	873 (51.7)	
**Income**				< 0.001*
Not enough	2275	1080 (47.5)	1195 (52.5)	
Enough	696	396 (56.9)	300 (43.1)	
**Having children**				0.004*
Yes	1470	770 (52.4)	700 (47.6)	
No	1501	706 (47.0)	795 (53.0)	
**Living with an elderly relative**				0.043*
Yes	1682	863 (51.3)	819 (48.7)	
No	1289	613 (47.6)	676 (52.4)	
**Number of family members (including the participant)**		5.0 ± 2.3	4.5 ± 2.0	< 0.001*
**Prepare/help in preparing food**				0.059
Yes	2072	1053 (50.8)	1019 (49.2)	
No	899	423 (47.1)	476 (52.9)	
**Chronic diseases**				0.432
Yes		1382 (49.9)	1389 (50.1)	
No		94 (47.0)	106 (53.0)	
**Self-assessment of food safety knowledge**				< 0.001*
Excellent		202 (49.0)	210 (51.0)	
Very good		513 (45.7)	610 (54.3)	
Good		585 (50.5)	573 (49.5)	
Weak		156 (62.7)	93 (37.3)	
Very weak		20 (69.0)	9 (31.0)	
**Source of food safety knowledge**				< 0.001*
Courses/workshops		73 (58.9)	51 (41.1)	
Social media platforms		235 (61.4)	148 (38.6)	
Family, acquaintances, and friends		177 (55.3)	143 (44.7)	
Internet		254 (52.6)	229 (47.4)	
Food safety professional		89 (49.7)	90 (50.3)	
Healthcare professional		96 (47.5)	106 (52.5)	
More than one source of the mentioned above		552 (43.1)	728 (56.9)	

### Multivariable analysis of factors associated with knowledge, attitudes, and practices

The results of the logistic regression analysis of predictors affecting good knowledge, attitudes, and practices of respondents towards the cholera outbreak are represented in Table 4. The significant predictors found to increase the probability of optimal knowledge were older age (OR = 1.03, 95% CI: 1.01–1.04, *p* < 0.001), higher education (OR = 1.42, 95% CI: 1.05–1.92, *p* = 0.024), living in outbreak countries (OR = 3.69, 95% CI; 2.95–4.61, *p* < 0.001) and working in the medical sector (OR = 2.59, 95% CI: 2.09–3.22, *p* < 0.001). Getting older (OR = 1.01, 95%CI: 1.0–1.03, *p* = 0.027) and working in the medical sectors (OR = 1.41, 95% CI: 1.13–1.75, *p* = 0.002) were retrieved to significantly increase the probability of good attitudes. For good food, water, and personal hygiene practices, being female (OR = 1.41, 95% CI: 1.16–1.71, *p* < 0.001) and having more than one source for food safety knowledge (OR = 1.63,95% CI: 1.04–2.54, *p* = 0.033) significantly increased the probability of optimal practices scores whereas working in medical sectors (OR = 0.79, 95% CI: 0.65–0.97, *p* = 0.022), and having an increased number of persons living in the house (OR = 0.93, 95% CI: 0.89–0.96, *p* < 0.001) significantly decreased the practice’s scores.

**Table 4:** Logistic regression analysis for predictors affecting knowledge, attitudes, and practices

Independent variables	*p*-value	OR	95% CI for OR	
			Lower	Upper
**Knowledge**				
**Age**	< 0.001*	1.026	1.014	1.038
**Higher education** ^**a**^	0.024*	1.418	1.048	1.918
**Presence of outbreak** ^**b**^	< 0.001*	3.687	2.950	4.609
**Medical sector** ^**c**^	< 0.001*	2.594	2.090	3.221
**Having children** ^**d**^	0.519	0.920	0.714	1.186
**Living with an elderly person** ^**d**^	0.322	0.889	0.704	1.122
**Attitude**				
**Age**	**0.027** ^*^	1.014	1.002	1.026
**Female sex** ^**e**^	0.112	0.835	0.668	1.043
**Married** ^**f**^	0.419	0.872	0.626	1.215
**Medical sector** ^**c**^	**0.002** ^*^	1.405	1.131	1.745
**Having children** ^**d**^	0.348	1.177	0.837	1.655
**Living with elder** ^**d**^	0.762	0.961	0.741	1.246
**Number of living persons in the same house**	0.287	0.972	0.921	1.025
**Presence of chronic disease** ^**d**^	0.403	0.805	0.485	1.337
**Practice**				
**Female sex** ^**e**^	0.001*	1.410	1.162	1.711
**Married** ^**f**^	0.379	0.890	0.687	1.154
**Medical sector** ^**c**^	0.022*	0.793	0.651	0.967
**Enough income** ^**g**^	0.071	0.817	0.656	1.018
**Having children** ^**d**^	0.060	0.777	0.598	1.010
**Living with an elderly person** ^**d**^	0.188	0.862	0.690	1.076
**Number of persons living in the same house**	< 0.001*	0.926	0.892	0.961
**Source of food safety knowledge** ^**h**^				
Courses/workshops	< 0.001*			
Social media platforms	0.173	0.701	0.421	1.169
Family, acquaintances, and friends	0.356	0.784	0.468	1.315
Internet	0.502	1.181	0.727	1.920
Food safety professional	0.856	0.948	0.534	1.683
Health care professional	0.073	1.633	0.954	2.795
More than one source of the mentioned above	0.033*	1.627	1.041	2.542

^a^ (ref); secondary or less, ^b^ (ref); cholera-free country, ^c^ (ref); outside the medical field, ^d^ (ref); No, ^e^ (ref); male, ^f^ (ref); un-married, ^g^ (ref); not enough, ^h^ (ref); courses/workshops

**Knowledge**,** attitudes**,** and self-reported practices regarding cholera among six MENA countries following cholera outbreaks in the region**

Salma A. Bekhit^1*^, Rayane Rafei^2^, Fatma Elnagar^3^, Omar Zain AL-Sakkaf^4^, Hussein Kamal Seif^5^, Dana Samardali^6^, Yara Turkmani Alabead^7^, Mohammed Osman Omer Sanosi^8^, Marwa Shawky Abdou^9^, Eman H. Elbanna^10^, Doaa Mahmoud Khalil^11^

## Discussion

This study highlights gaps in cholera-related knowledge, attitudes, and practices (KAP) across six MENA countries, including outbreak-affected and cholera-free countries, based on the recent updates reported by the WHO and European Centre for Disease Prevention and Control (ECDC) [12, 29]. Our results reveal differences in knowledge, attitudes, and practices, particularly in cholera-free countries, emphasizing the critical need for proactive measures and country-specific interventions to enhance cholera prevention, control, and community resilience.

### Cholera knowledge

In the present study, most participants heard about cholera and responded correctly to the questions about the cause, susceptible populations, modes of transmission, and symptoms. Similar levels of good cholera knowledge were reported by studies conducted in Lebanon [26], South Africa [25], Tanzania [30], Mozambique [31], Haiti [28], and vaccinated areas in the Solomon Islands [32]. Conversely, poor knowledge about cholera transmission routes and prevention methods was observed in studies from Yemen [27, 33], Saudi Arabia [34], and Bangladesh [6]. While most participants in this study correctly identified the modes of cholera transmission correctly, gaps in knowledge were evident, with 56.2% were unaware that eating raw or undercooked fish and seafood can transmit cholera, a well-documented source of infection as reported by the Centers for Disease Control and Prevention (CDC) [35].

Moreover, there was limited awareness of cholera’s ability to spread in overcrowded settings with poor sanitation and hygiene, with approximately 55% of participants unaware of this transmission risk [36]., especially since some parts of the studied countries have high population densities. Although respondents were familiar with common symptoms like watery diarrhea (88.2%) and severe dehydration (88.7%), only few (5.1%) recognized the asymptomatic nature of most cholera infections, which contributes to silent transmission and complicates outbreak control efforts. This lack of awareness is critical, as asymptomatic carriers can shed *V. cholerae* in feces for up to 10 days, contributing to the underreporting of cases and the misclassification of cholera-related deaths [37, 38]. Such asymptomatic cases represent a significant challenge for cholera control and eradication efforts, as only 5–10% of global cases are reported to the WHO [37]. Thus, enhancing public knowledge about asymptomatic transmission is essential for mitigating outbreaks. Furthermore, given the endemic nature of cholera in the MENA region, public healthcare systems should adopt regular proactive surveillance measures, similar to the effective strategies implemented by Saudi Arabia during the Hajj season [39].

Knowledge about the route of cholera vaccine administration was suboptimal, with 61.1% of participants unaware of it, consistent with findings from Bangladesh and Yemen, which also reported similarly low awareness [6, 27, 33]. This gap can be attributed to several factors, including the significant global paucity of cholera vaccines, the absence of health education campaigns, together with the high skepticism and hesitancy among the public in the Middle East region regarding vaccines following debates over COVID-19 vaccines [4, 40]. Participants from cholera-outbreak countries (61.8%) generally demonstrated better overall cholera knowledge compared to those in cholera-free countries (30.3%). Similar findings from previous studies linked greater awareness to outbreaks, likely due to increased curiosity of people living in outbreak-affected countries and intensified public awareness campaigns by local authorities and humanitarian organizations [25, 28, 34].

A significantly higher knowledge was associated with a higher mean age of participants, having a university and above degree, being employed, working in the medical sector, having children, and not living with elderly relatives. A similar finding to our study from Saudi Arabia stated that sex was not significantly associated with good cholera knowledge [34]. In contrast to our results, recent studies from Lebanon and Saudi Arabia linked better knowledge to female participants [26, 34, 41]. In line with our findings, studies from Lebanon and Saudi Arabia reported that higher education was significantly linked to good knowledge [26, 34]. Expectingly, participants working in the medical field had better overall knowledge about cholera, but this knowledge was still inadequate, as 35.8% had poor knowledge of cholera. Workers in the medical sector are the first line of defense against any infectious disease; their role was clearly evident lately during the COVID-19 pandemic. Poor knowledge among this group could delay diagnosis and facilitate the spread of infection [42]. Therefore, it is essential to enhance the capacities of workers in the medical sector and provide them with continuous educational training. Interestingly, having children was significantly associated with good cholera knowledge, likely due to the mothers’ care and worries about their children, which is considered a positive finding given that children are a vulnerable group and more prone to infection. This finding emphasizes the importance of targeting mothers in educational campaigns to further enhance public awareness and prevention efforts.

Logistic regression revealed that optimal cholera knowledge was significantly associated with older age, higher education, living in outbreak countries, and working in the medical sector. Knowledge was also strongly linked to positive attitudes and preventive practices, suggesting that the dissemination of good knowledge will improve attitudes and practices towards cholera and thus help in containing the outbreak. Addressing existing knowledge gaps through targeted public health education is essential to enhance community resilience and cholera prevention efforts.

### Attitudes towards cholera and oral cholera vaccine

Our results showed that most participants (67.3%) demonstrated good attitudes toward cholera, with many recognizing its severity and the increased risk of infection when traveling to outbreak countries. Similar results were reported by studies among the Lebanese and Saudi Arabian populations, who were attentive to the infectious nature of cholera and the possibility of being lethal if not treated [26, 34]. Although two-thirds of the respondents were aware of the protective role of the vaccines against cholera, most were suspicious about the side effects that may result after vaccination. These results can be elucidated by the recent claims about COVID-19 vaccines’ side effects. Fortunately, the fear of the cholera infection and its consequences outweighs the study participants’ fear of the oral cholera vaccine side effects. Several KAP studies demonstrated positive attitudes of their participants towards cholera vaccination [6, 26, 30, 31, 34, 43].

Attitudes were significantly influenced by several factors, such as age, sex, education, marital status, employment, field of work, residence, having children, living with elders, number of family members, and chronic diseases. Similarly, other studies showed that better attitudes were associated with older age, being married, working in the medical field, and good knowledge [26]. These associations emphasize the importance of focusing on sociodemographic variations in public health strategies to promote favorable attitudes toward cholera prevention and vaccination efforts.

### Self-reported practices related to cholera

The current study revealed that nearly half of the participants generally exhibited good food, water, and personal hygiene practices. Females were likely to have better practices than males; this could be justified by the fact that females are more concerned about the health of themselves and their families, and thus, they are more inclined to have better hygienic practices. Previous KAP studies reported the superiority of female practices [31, 34, 41]. Being unmarried, having no children, unemployed or retired, without enough income, and not living with an elderly person were associated with good practices. Surprisingly, working in the medical field was associated with poor practices, despite their good knowledge and attitude score related to cholera. This highlights the need for targeted health education to promote proper practices among medical professionals. A potential explanation is response bias, where non-medical participants may overreport or misrepresent their practices, leading to higher reported scores.

We found that a higher number of family members was associated with poor practices, aligning with a recent Lebanese study that calculated the crowding index, reporting that a higher house crowding index was negatively associated with better preventive practices [41]. Larger families and higher crowding indices usually indicate a low socioeconomic class, which may be correlated with poor health education, lack of access to safe water, proper sanitation facilities, and poor hygiene [44].

Social media, though widely accessible, was associated with poorer cholera practices, highlighting its dual role as a platform for disseminating health and food safety information while also serving as a potential source of misinformation [41, 45]. Therefore, public health systems in the MENA region must make use of social media platforms to build awareness about any health crisis and proper hygienic practices and disseminate accurate information. Conversely, participants who relied on multiple credible sources, such as healthcare and food safety professionals, exhibited better practices, highlighting the importance of reliance on diverse and reliable information.

Water is a critical vector for cholera transmission, thus management of water sources in the MENA region is essential for cholera control. The water sources vary between cholera-outbreak countries and free-cholera countries; for instance, private wells are more used in outbreak settings (74.6%) rather than free-cholera areas (25.4%), with Lebanon being the biggest consumer of water from wells (Additional file 1: Table S8). Unfortunately, groundwater quality in Lebanon has deteriorated due to anthropogenic pollution and over-abstraction with much evidence of microbiological contamination [46] and subsequently the potential role of water in being a vector of cholera transmission. Electricity outages further exacerbate the issue, forcing reliance on unregulated water sources, and emphasizing the need for water testing and treatment during outbreaks. A considerable portion of the population (27.8%) does not treat or test their drinking water, perhaps due to a perceived false sense of its safety, as reported by other studies [27, 33]. Effective cholera prevention necessitates a comprehensive strategy that integrates regular water testing, public awareness campaigns, and collaboration among stakeholders to secure clean and safe water sources.

The major strengths of the current study are its large sample size, the diverse populations relative to previous studies, and its time that coincides with the existing cholera outbreaks in multiple Arab countries in the MENA region. However, one limitation is the potential for self-reporting bias, as participants may provide socially desirable responses or inaccurately recall information. To mitigate this bias, we conducted a pilot test of the questionnaire, ensured the anonymity and confidentiality of responses, and utilized a validated instrument. Furthermore, the survey was conducted online; thus, only people with internet access or knowledge could participate, which may limit the generalizability of the results to all countries in the MENA region. The war restrictions and the blockage of websites further hindered better sampling. Consequently, when feasible, future studies should consider these limitations to further validate our results.

## Conclusions

This study aimed to fill the knowledge gap in the literature on KAP related to cholera in both outbreak and non-outbreak countries in the MENA region. The study revealed that nearly half of the participants reported a satisfying level of knowledge and good practices, while two-thirds of the participants had a positive attitude towards cholera. Lower education levels and living in cholera-free countries were found to be associated with poor knowledge levels. Although medical sector participants reported excellent levels of knowledge, this knowledge was not reflected in their practices. It is therefore important to provide people, including workers in the medical field, living in the highly vulnerable countries in the MENA region with education programs and oral cholera vaccines. Public health authorities, policymakers, and international organizations should take part in implementing preventive plans, providing hygiene supplies, and investing in the development of infrastructure. A special focus on food, water, and personal hygiene education and ensuring the proper application of this knowledge with the provision of alternative solutions, in case of absence of some of the basic needs, is vital to prevent and eradicate not only cholera but many other infectious diseases.

## Electronic supplementary material

Below is the link to the electronic supplementary material.

Supplementary Material 1

Supplementary Material 2

## Data Availability

The raw data analyzed during the current study are available as a supplementary file.

## Abbreviations

CDC: Centers for Disease Control and Prevention
CI: Confidence interval
COVID-19: Coronavirus Disease 2019
ECDC: European Centre for Disease Prevention and Control
KAP: Knowledge, Attitude and Practices
MENA: Middle East and North Africa
OCV: Oral Cholera Vaccines
OR: Odds ratio
ORS: Oral Rehydration Solutions
V. cholerae: Vibrio cholerae
WHO: World Health Organization
