# Supplementary material for: Knowledge, attitudes, and self-reported practices regarding cholera among six MENA countries following cholera outbreaks in the region
Source: BMC Public Health. 2025 Feb 18;25:674. doi: 10.1186/s12889-025-21731-6 (PMC11837374; doi:10.1186/s12889-025-21731-6)
Supplement: Supplementary file 1 — Supplementary Material 1 [file 12889_2025_21731_MOESM1_ESM.docx]

**Additional File 1 for:**

**Knowledge, attitudes, and self-reported practices regarding cholera among six MENA countries following cholera outbreaks in the region**

Salma A. Bekhit^1*^, Rayane Rafei^2^, Fatma Elnagar^3^, Omar Zain AL-Sakkaf^4^, Hussein Kamal Seif^5^, Dana Samardali^6^, Yara Turkmani Alabead^7^, Mohammed Osman Omer Sanosi^8^, Marwa Shawky Abdou^9^, Eman H. Elbanna^10^, Doaa Mahmoud Khalil^11^

**Supplementary files**

**Table S1 Baseline characteristics of the study participants (N=2971).**

| **Characteristics** | **N (%)^1^** |
| --- | --- |
| **Sex** |  |
| Male | 1115 (37.5) |
| Female | 1856 (62.5) |
| **Age** |  |
| Min. – Max. | 18.0 – 66.0 |
| Mean ± SD | 34.8 ± 12.3 |
| **Marital status** |  |
| Single | 1071 (36.1) |
| Married | 1661 (55.9) |
| Widowed | 161 (5.4) |
| Divorced | 78 (2.6) |
| **Residence** |  |
| Yemen | 625 (21.0) |
| Lebanon | 570 (19.2) |
| Egypt | 524 (17.6) |
| Jordan | 517 (17.4) |
| Syria | 494 (16.6) |
| Sudan | 241 (8.1) |
| **Nationality** |  |
| Yemeni | 638 (21.5) |
| Lebanese | 548 (18.4) |
| Syrian | 525 (17.7) |
| Egyptian | 504 (17.0) |
| Jordanian | 490 (16.5) |
| Sudanese | 249 (8.4) |
| Others | 17 (0.6) |
| **Educational level** |  |
| Secondary education or below | 629 (21.2) |
| University education | 1657 (55.8) |
| Postgraduate studies | 685 (23.1) |
| **Employment status** |  |
| Employed | 1878 (63.2) |
| Unemployed | 993 (33.4) |
| Retired | 100 (3.4) |
| **Working field (n= 1878)** |  |
| Non-medical | 1102 (58.7) |
| Medical | 776 (41.3) |
| **Income** |  |
| Not enough and on a loan | 462 (15.6) |
| Not enough | 956 (32.2) |
| Enough | 1319 (44.4) |
| Enough and saving | 234 (7.9) |
| **Having children** | 1470 (49.5) |
| **Living with an elderly relative** | 1682 (56.6) |
| **Median number of family members (including the participant) with the range** | 5 (1-11) |
| **Prepare/help in preparing food** | 2072 (69.7) |
| **Self-assessment of food safety knowledge** |  |
| Excellent | 412 (13.9) |
| Very good | 1123 (37.8) |
| Good | 1158 (39.0) |
| Weak | 249 (8.4) |
| Very weak | 29 (1.0) |
| **Source of food safety knowledge** |  |
| Courses/workshops | 124 (4.2) |
| Social media platforms | 383 (12.9) |
| Family, acquaintances, and friends | 320 (10.8) |
| Internet | 483 (16.3) |
| Food safety professional | 179 (6.0) |
| Healthcare professional | 202 (6.8) |
| More than one source of the mentioned above | 1280 (43.1) |
| **Chronic diseases** | 1151 (38.7) |

^1^ This column represents the number and the percentage enclosed between parentheses unless other measures were specified.

**Table S2 Participants’ information source and previous experience with cholera (N= 2971).**

| **Items** | **N (%)** |
| --- | --- |
| **Heard about cholera disease** | 2536 (85.4) |
| **Heard about the cholera outbreaks in some Arab countries (n=2536)** | 2179 (85.9) |
| **Source of information about cholera outbreaks (n=2179)** |  |
| Personal experience | 56 (2.6) |
| Healthcare provider | 235 (10.8) |
| Social media | 535 (24.6) |
| TV or Radio | 220 (10.1) |
| Family members, acquittances, neighbors, or friends | 58 (2.7) |
| Banners, posters, or leaflets | 28 (1.3) |
| Loudspeakers of (Mosques, Churches, Schools, and others) | 3 (0.1) |
| Religious or political leaders | 1 (0.0) |
| More than one source | 1043 (47.9) |
| **Infection of the participants with cholera during the outbreak (n=2179)** |  |
| Yes (confirmed) | 42 (1.9) |
| No | 52 (2.4) |
| Not sure | 2085 (95.7) |
| **Need for hospitalization (n= 42)** | 5 (11.9) |
| **Infection of the participants’ relatives with cholera during the outbreak (n= 2179)** |  |
| Yes (confirmed) | 160 (7.3) |
| No | 1835 (84.2) |
| Not sure/ Do not know | 184 (8.4) |
| **Need of the participants’ relatives for hospitalization (n= 160)** |  |
| Yes | 61 (38.1) |
| No | 86 (53.8) |
| Do not know | 13 (8.1) |
| **Mortality of relatives due to cholera (n= 2179)** |  |
| Yes | 29 (1.3) |
| No | 2089 (95.9) |
| Do not know | 61 (2.8) |
| **What will you do if you or one of your family members get infected with cholera? (n= 2536)** |  |
| Herbal treatment or traditional medicine | 22 (0.7) |
| Prepare an oral rehydration solution at home | 235 (7.9) |
| Going to the health center/hospital/clinic and taking prescribed medication | 1570 (52.8) |
| Not sure/ Do not know | 60 (2.0) |
| More than one action (of those mentioned above) | 649 (21.8) |

**Table S3 Knowledge and attitudes of participants toward cholera (N=2536)**

|  | **Response** | **N (%)** |
| --- | --- | --- |
| **Knowledge (mean score= 13.7 ± 4.1)** | | |
| **General knowledge** |  |  |
| Affect all ages | Correct | 2263 (89.2) |
|  | Incorrect | 273 (10.8) |
| Causative agent | Correct | 1614 (63.6) |
|  | Incorrect | 922 (36.4) |
| Most of the cases are asymptomatic | Correct | 130 (5.1) |
|  | Incorrect | 2406 (94.9) |
| Route of cholera vaccine administration | Correct | 986 (38.9) |
|  | Incorrect | 1550 (61.1) |
| **Modes of transmission** |  |  |
| Raw or undercooked fish and seafood | Correct | 1111 (43.8) |
|  | Incorrect | 1425 (56.2) |
| Contaminated water | Correct | 2415 (95.2) |
|  | Incorrect | 121 (4.8) |
| Patients’ blood | Correct | 1411 (55.6) |
|  | Incorrect | 1125 (44.4) |
| Contaminated unwashed vegetables and fruits | Correct | 2247 (88.6) |
|  | Incorrect | 289 (11.4) |
| Disruption of the water distribution system | Correct | 2196 (86.6) |
|  | Incorrect | 340 (13.4) |
| Sexual contact | Correct | 1682 (66.3) |
|  | Incorrect | 854 (33.7) |
| Contaminated street-vended food and beverages | Correct | 2147 (84.7) |
|  | Incorrect | 389 (15.3) |
| Pets | Correct | 1248 (49.2) |
|  | Incorrect | 1288 (50.8) |
| Polluted air | Correct | 1684 (66.4) |
|  | Incorrect | 852 (33.6) |
| Uncovered food potentially contaminated by insects and flies | Correct | 1906 (75.2) |
|  | Incorrect | 630 (24.8) |
| Overcrowded places | Correct | 1153 (45.5) |
|  | Incorrect | 1383 (54.5) |
| **Symptoms** |  |  |
| Respiratory symptoms | Correct | 1460 (57.6) |
|  | Incorrect | 1074 (42.4) |
| Watery diarrhea | Correct | 2234 (88.2) |
|  | Incorrect | 300 (11.8) |
| Nausea and vomiting | Correct | 1967 (77.7) |
|  | Incorrect | 566 (22.3) |
| Severe dehydration | Correct | 2095 (82.7) |
|  | Incorrect | 438 (17.3) |
| Inflamed joints | Correct | 1420 (56.1) |
|  | Incorrect | 1113 (43.9) |
| Itching | Correct | 1379 (54.5) |
|  | Incorrect | 1153 (45.5) |
| **Attitudes (mean score= 2.4 ± 0.3)** | | |
| I believe that cholera is a severe health problem that may cause death | Agree | 2262 (89.2) |
|  | Neutral | 216 (8.5) |
|  | Disagree | 58 (2.3) |
| I believe that the cholera vaccine can protect me from cholera | Agree | 1795 (70.8) |
|  | Neutral | 610 (24.1) |
|  | Disagree | 131 (5.2) |
| I believe that the vaccine for cholera may cause dangerous side effects | Agree | 926 (36.5) |
|  | Neutral | 1179 (46.5) |
|  | Disagree | 431 (17.0) |
| I believe I am susceptible to getting infected with cholera | Agree | 1199 (47.3) |
|  | Neutral | 797 (31.4) |
|  | Disagree | 540 (21.3) |
| I believe that I have a role in the prevention of the spread of cholera to the community | Agree | 723 (28.5) |
|  | Neutral | 805 (31.7) |
|  | Disagree | 1008 (39.7) |
| I believe that travelling to an area having an outbreak of cholera can increase the risk of contracting cholera | Agree | 1417 (55.9) |
|  | Neutral | 665 (26.2) |
|  | Disagree | 454 (17.9) |

**Table S4 Hand hygiene, food, and water safety practices of the study participants**

| **Practice items** | **Study sample (N=2971)** | | | | |
| --- | --- | --- | --- | --- | --- |
|  | **Never** | **Rarely** | **Sometimes** | **Often** | **Always** |
| **Hand hygiene practices (mean score = 4.1 ± 1.0)** | | | | | |
| Regular hand washing with clean water and soap | 233 (7.8) | 51 (1.7) | 221 (7.4) | 977 (32.9) | 1489 (50.1) |
| Hand washing with clean water and soap after using the toilet | 234 (7.9) | 73 (2.5) | 156 (5.3) | 889 (29.9) | 1619 (54.5) |
| Hand washing with clean water and soap before eating | 1. (10.6) | 151 (5.1) | 1. (11.6) | 952 (32.0) | 1207 (40.6) |
| Hand washing with clean water and soap before preparing food | 258 (8.7) | 106 (3.6) | 266 (9.0) | 974 (32.8) | 1367 (46.0) |
| Hand washing with clean water and soap after preparing raw food | 237 (8.0) | 120 (4.0) | 281 (9.5) | 947 (31.9) | 1386 (46.7) |
| **Food and water safety Practices (mean score = 3.9 ± 1.0)** | | | | | |
| Clean and sanitize food contact surfaces before and after using | 251 (8.4) | 162 (5.5) | 433 (14.6) | 960 (32.3) | 1165 (39.2) |
| Food is served hot | 229 (7.7) | 118 (4.0) | 438 (14.7) | 1062 (35.7) | 1124 (37.8) |
| Covering food to protect it from flies and other insects | 267 (9.0) | 73 (2.5) | 182 (6.1) | 1003 (33.8) | 1446 (48.7) |
| Storing food containers in a clean and dry place | 275 (9.3) | 88 (3.0) | 194 (6.5) | 968 (32.6) | 1446 (48.7) |
| Storing food to be prepared and leftovers in the refrigerator at a temperature of 5°C or less | 273 (9.2) | 146 (4.9) | 361 (12.2) | 982 (33.1) | 1209 (40.7) |
| Cooking food thoroughly to 70°C or above | 245 (8.2) | 110 (3.7) | 269 (9.1) | 995 (33.5) | 1352 (45.5) |
| Using separate cutting boards for raw and cooked foods. | 313 (10.5) | 276 (9.3) | 484 (16.3) | 820 (27.6) | 1078 (36.3) |
| Using the same source of water that is used for drinking to cook food | 395 (13.3) | 188 (6.3) | 326 (11.0) | 884 (29.8) | 1178 (39.6) |
| Drinking water from a clean and safe source | 395 (13.3) | 188 (6.3) | 326 (11.0) | 884 (29.8) | 1178 (39.6) |
| Treating the water to make sure it is clean | 270 (9.1) | 116 (3.9) | 219 (7.4) | 977 (32.9) | 1389 (46.8) |
| **Total practice score (Mean± SD)** | **3.9 ± 1.0** | | | | |

**Table S5 Sources, methods of treatment, and quality testing of the drinking water as reported by the study participants**

|  | **Study sample (No.=2971)** |
| --- | --- |
| **Source of water** |  |
| Public water network (faucet or tap at home or public) | 1102 (37.1) |
| Private well (artesian wells in the house) | 134 (4.5) |
| Public well | 94 (3.2) |
| Water trucks | 134 (4.5) |
| Water tank | 51 (1.7) |
| Surface water | 23 (0.8) |
| Collection of rainwater | 11 (0.4) |
| Bottled water | 230 (7.7) |
| More than one source | 1192 (40.1) |
| **Methods used for water treatment** |  |
| Do not treat water | 826 (27.8) |
| Boiling | 414 (13.9) |
| Subject the water to sunlight (solar disinfection) | 74 (2.5) |
| Chlorination | 233 (7.8) |
| Filtration using a clean cloth | 110 (3.7) |
| Sand or gravel water filtration | 29 (1.0) |
| Adding alum | 18 (0.6) |
| Use a water filter cartridge at home | 385 (13.0) |
| Do not know | 112 (3.8) |
| More than one of the above-mentioned methods | 770 (25.9) |
| **Water quality testing** | 583 (19.6) |

**Table S6: Comparison of water sources between cholera outbreak settings and cholera-free settings**

| **Source of water** | **Outbreak** | | **Total** |
| --- | --- | --- | --- |
|  | **No** | **Yes** |  |
| Public water network (daily/faucet or tap at home or public) | 537 | 565 | 1102 |
|  | 48.7% | 51.3% | 100.0% |
| Private well (artesian wells in the house) | 34 | 100 | 134 |
|  | 25.4% | 74.6% | 100.0% |
| Public well | 51 | 43 | 94 |
|  | 54.3% | 45.7% | 100.0% |
| By white / carts with a tank or tanks containing clean water distributed by the responsible authorities | 51 | 83 | 134 |
|  | 38.1% | 61.9% | 100.0% |
| A tank or a dam | 33 | 18 | 51 |
|  | 64.7% | 35.3% | 100.0% |
| From the valley | 13 | 5 | 18 |
|  | 72.2% | 27.8% | 100.0% |
| Collection of rainwater | 8 | 3 | 11 |
|  | 72.7% | 27.3% | 100.0% |
| In a water pond | 5 | 0 | 5 |
|  | 100.0% | 0.0% | 100.0% |
| Bottled water (mineral water) sold through companies licensed by the responsible authorities | 77 | 153 | 230 |
|  | 33.5% | 66.5% | 100.0% |
| More than one source | 473 | 719 | 1192 |
|  | 39.7% | 60.3% | 100.0% |
| **Total** | 1282 | 1689 | 2971 |
|  | 43.2% | 56.8% | 100.0% |

**Table S7: Differences between the methods of water treatment between cholera outbreak settings and cholera-free settings**

| **How do you clean water** | **Outbreak** | | **Total** |
| --- | --- | --- | --- |
|  | **no** | **Yes** |  |
| No | 256_a_ | 570_b_ | 826 |
|  | 31.0% | 69.0% | 100.0% |
| By boiling | 138_a_ | 276_b_ | 414 |
|  | 33.3% | 66.7% | 100.0% |
| Save water in the hot sun | 64_a_ | 10_b_ | 74 |
|  | 86.5% | 13.5% | 100.0% |
| Disinfection by adding chlorine | 84_a_ | 149_b_ | 233 |
|  | 36.1% | 63.9% | 100.0% |
| Filtration or filtration with a cloth | 68_a_ | 42_b_ | 110 |
|  | 61.8% | 38.2% | 100.0% |
| Sand filtration (shallow borehole) | 23_a_ | 6_b_ | 29 |
|  | 79.3% | 20.7% | 100.0% |
| Cleansing by adding alum | 16_a_ | 2_b_ | 18 |
|  | 88.9% | 11.1% | 100.0% |
| Use a filter or ceramic filter / beuzand (filter or filter cartridge) | 219_a_ | 166_b_ | 385 |
|  | 56.9% | 43.1% | 100.0% |
| Don’t know | 43_a_ | 69_a_ | 112 |
|  | 38.4% | 61.6% | 100.0% |
| More than one method | 371_a_ | 399_b_ | 770 |
|  | 48.2% | 51.8% | 100.0% |
| **Total** | 1282 | 1689 | 2971 |
|  | 43.2% | 56.8% | 100.0% |

**Table S8: Comparison of water sources between the different studied countries**

| Source of water | The residence country | | | | | | | Total |
| --- | --- | --- | --- | --- | --- | --- | --- | --- |
|  | Egypt | Sudan | Jordan | Syria | Lebanon | Yemen |  | |
| Public water network (daily/faucet or tap at home or public) | 349 | 175 | 13 | 241 | 102 | 222 | 1102 | |
|  | 31.7% | 15.9% | 1.2% | 21.9% | 9.3% | 20.1% | 100.0% | |
| Private well (artesian wells in the house) | 4 | 2 | 28 | 9 | 82 | 9 | 134 | |
|  | 3.0% | 1.5% | 20.9% | 6.7% | 61.2% | 6.7% | 100.0% | |
| Public well | 1 | 7 | 43 | 3 | 22 | 18 | 94 | |
|  | 1.1% | 7.4% | 45.7% | 3.2% | 23.4% | 19.1% | 100.0% | |
| By white / carts with a tank or tanks containing clean water distributed by the responsible authorities | 4 | 7 | 40 | 11 | 11 | 61 | 134 | |
|  | 3.0% | 5.2% | 29.9% | 8.2% | 8.2% | 45.5% | 100.0% | |
| A tank or a dam | 4 | 2 | 27 | 0 | 10 | 8 | 51 | |
|  | 7.8% | 3.9% | 52.9% | 0.0% | 19.6% | 15.7% | 100.0% | |
| From the valley | 0 | 1 | 12 | 1 | 2 | 2 | 18 | |
|  | 0.0% | 5.6% | 66.7% | 5.6% | 11.1% | 11.1% | 100.0% | |
| Collection of rainwater | 0 | 0 | 8 | 0 | 1 | 2 | 11 | |
|  | 0.0% | 0.0% | 72.7% | 0.0% | 9.1% | 18.2% | 100.0% | |
| In a water pond | 0 | 1 | 4 | 0 | 0 | 0 | 5 | |
|  | 0.0% | 20.0% | 80.0% | 0.0% | 0.0% | 0.0% | 100.0% | |
| Bottled water (mineral water) sold through companies licensed by the responsible authorities | 56 | 7 | 14 | 17 | 90 | 46 | 230 | |
|  | 24.3% | 3.0% | 6.1% | 7.4% | 39.1% | 20.0% | 100.0% | |
| More than one source | 106 | 39 | 328 | 212 | 250 | 257 | 1192 | |
|  | 8.9% | 3.3% | 27.5% | 17.8% | 21.0% | 21.6% | 100.0% | |
| Total | 524 | 241 | 517 | 494 | 570 | 625 | 2971 | |
|  | 17.6% | 8.1% | 17.4% | 16.6% | 19.2% | 21.0% | 100.0% | |

**Table S9: Comparison of methods of water cleaning between the different studied countries**

| How do you clean water | **The residence country** | | | | | | Total |
| --- | --- | --- | --- | --- | --- | --- | --- |
|  | **Egypt** | **Sudan** | **Jordan** | **Syria** | **Lebanon** | **Yemen** |  |
| No | 153_a_ | 86_a, b_ | 17_c_ | 207_b_ | 169_a_ | 194_a_ | 826 |
|  | 18.5% | 10.4% | 2.1% | 25.1% | 20.5% | 23.5% | 100.0% |
| By boiling | 27_a_ | 44_b, c, d_ | 67_d_ | 122_c_ | 85_b, d_ | 69_b, d_ | 414 |
|  | 6.5% | 10.6% | 16.2% | 29.5% | 20.5% | 16.7% | 100.0% |
| Save water in the hot sun | 1_a_ | 1_a_ | 62_b_ | 1_a_ | 4_a_ | 5_a_ | 74 |
|  | 1.4% | 1.4% | 83.8% | 1.4% | 5.4% | 6.8% | 100.0% |
| Disinfection by adding chlorine | 4_a_ | 16_b_ | 64_b, c_ | 5_a_ | 82_c_ | 62_b, c_ | 233 |
|  | 1.7% | 6.9% | 27.5% | 2.1% | 35.2% | 26.6% | 100.0% |
| Filtration or filtration with a cloth | 35_a_ | 5_a, b, c_ | 28_a, c_ | 6_b_ | 14_b, c_ | 22_a, b, c_ | 110 |
|  | 31.8% | 4.5% | 25.5% | 5.5% | 12.7% | 20.0% | 100.0% |
| Sand filtration (shallow borehole) | 1_a_ | 0_a_ | 22_b_ | 4_a_ | 1_a_ | 1_a_ | 29 |
|  | 3.4% | 0.0% | 75.9% | 13.8% | 3.4% | 3.4% | 100.0% |
| Cleansing by adding alum | 1_a_ | 3_a, b_ | 12_b_ | 0_a_ | 1_a_ | 1_a_ | 18 |
|  | 5.6% | 16.7% | 66.7% | 0.0% | 5.6% | 5.6% | 100.0% |
| Use a filter or ceramic filter / beuzand (filter or filter cartridge) | 194_a_ | 20_b, c, d_ | 5_e_ | 69_d_ | 29_c_ | 68_b, d_ | 385 |
|  | 50.4% | 5.2% | 1.3% | 17.9% | 7.5% | 17.7% | 100.0% |
| Don’t know | 23_a_ | 15_a_ | 5_b_ | 11_a, b_ | 27_a_ | 31_a_ | 112 |
|  | 20.5% | 13.4% | 4.5% | 9.8% | 24.1% | 27.7% | 100.0% |
| More than one method | 85_a_ | 51_a, b_ | 235_c_ | 69_a_ | 158_b_ | 172_b_ | 770 |
|  | 11.0% | 6.6% | 30.5% | 9.0% | 20.5% | 22.3% | 100.0% |
| Total | 524 | 241 | 517 | 494 | 570 | 625 | 2971 |
|  | 17.6% | 8.1% | 17.4% | 16.6% | 19.2% | 21.0% | 100.0% |


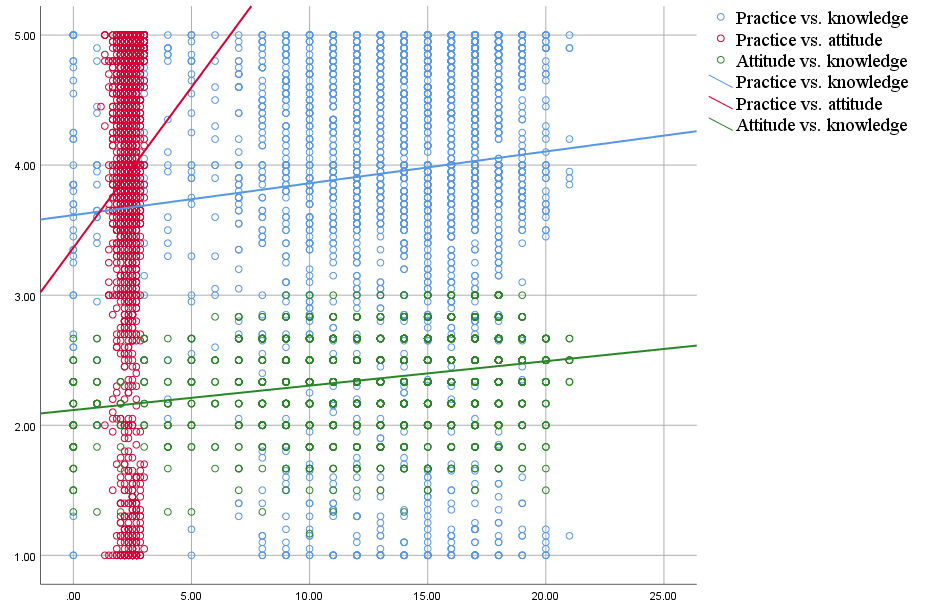


**Figure S1 Correlation between knowledge, attitudes, and practice scores**
